# Supplementary material for: Are carbon emissions trading and green financial instruments synergistic? -Comprehensive quantitative research based on content analysis
Source: PLoS One. 2024 Mar 7;19(3):e0298601. doi: 10.1371/journal.pone.0298601 (PMC10919601; doi:10.1371/journal.pone.0298601)
Supplement: S1 Data — (ZIP) [file pone.0298601.s001.zip › Data/地级市Z_F工作报告.zip!/║┌┴·╜¡/╦1⁄2╤╝╔╜╩╨/└┤╫╘╣1⁄2╓┌║┼í╛┬φ┐╦╩2╛▌═°í┐_2018.docx]

各位代表：

现在，我代表市人民政府，向大会报告工作，请予审议，并请市政协委员提出意见。

一、2017年工作回顾

2017年，我们以习近平总书记系列重要讲话精神特别是对我省重要讲话精神为统领，深入学习贯彻落实党的十九大、省十二次党代会和市十一次党代会精神，在市委的正确领导下，围绕市十六届人大一次会议确定的目标任务，牢牢把握稳中求进工作总基调，紧紧扭住转型发展这条主线，以创新驱动为引领，全力推进稳增长、促改革、调结构、惠民生各项工作，凝神聚力，攻坚克难，实干担当，取得了经济平稳向好、转型步伐加快、改革纵深推进、民生持续改善、社会和谐稳定的新成效。预计全市实现地区生产总值468亿元，同比增长5%；规模以上工业增加值53.7亿元，增长3.3%；固定资产投资143亿元，增长10%；公共财政预算收入22.1亿元，增长8.6%；社会消费品零售总额139亿元，增长8%；城镇和农村常住居民人均可支配收入分别达到23760元、13820元，均增长6%。

（一）坚定不移转方式调结构，经济发展速度和效益稳中向好

产业项目建设取得显著成效。全市开复工投资500万元以上产业项目146个、竣工投产62个、完成投资75亿元；招商引资签约项目150个，到位资金120亿元；策划生成项目170个。强力推进“粮头食尾”、“农头工尾”建设，粮食和绿色食品深加工产业项目集群初步形成，宝清县万里润达150万吨玉米深加工、集贤县鸿展30万吨和威克特10万吨特优食用酒精、友谊县北大荒龙麦30万吨玉米特强粉等项目开工建设或建成投产，全省布局15个玉米燃料乙醇项目中，市经开区、集贤县和宝清县3个项目进入前10名。以建设现代煤化工基地为抓手，加快推进“煤头化尾”、“煤头电尾”建设，现代煤化工产业园区完成规划编制，龙煤天泰芳烃项目具备甲醇生产条件，神华国能宝清电厂加快建设。坚定走好“探采加一体化”石墨产业发展路径，依法取得西山、羊鼻山探矿权，岭东区中双石墨采选及深加工项目开工建设。加快发展畜牧养殖及深加工产业，宝清县中顺万头肉牛养殖一期、尖山区谷实农牧生猪放养项目建成，集贤县大北农百万头生猪一体化项目扎实推进。电商产业提档升级，市县区电商产业园投入使用，发挥了重要引领和带动作用；新建电商园、创客空间34个，电商平台12个；举办了第二届中国·双鸭山微商峰会和中国微商健康规范发展座谈会。全市电商微商从业人员达到万人，快递业务收入比上年增长31%。经济运行质量明显提升。各项经济指标全面增长，特别是工业经济扭转连续4年负增长态势，实现了正增长，全市规模以上工业企业利润扭亏增盈9.4亿元。新培育临规工业企业31户、盘活停产企业22户。非公经济实现较快增长，占经济总量比重达到50%。重点企业效益大幅增长，双矿公司扭转连续5年亏损局面，扭亏增盈10.8亿元；建龙钢铁利润增长21倍。“三去一降一补”取得积极成效，化解煤炭过剩产能24万吨，商品房去库存81.1万平方米，粮食去库存261.9万吨。新旧动能转换步伐加快。瞄准既有国内总需求空间、又有我市供给优势的新增长领域，深度谋划和推动了现代煤化工、粮食和绿色食品深加工、石墨及新材料、旅游、电商、畜牧、特色种植七个新增长领域，正逐步成为拉动经济增长的新动能。坚持把转型发展的基点放在创新上，制定了促进科技成果产业化实施细则和考核办法，成立了科技成果产业化创新发展联盟，广泛与省内外高校、科研院所建立合作关系，已与中科院、哈工大等8所高校、科研院所签订了市校(院、所)合作协议，签订科技成果转化、项目合作协议46个，一批科技成果已在双转化落地。

一年来，我们以提质增效为核心，全力做好“三篇大文章”，优存量、扩增量，加快新旧动能转换，推动产业转型升级，经济发展呈现出稳中有进、进中向好的良好态势。

（上接一版）

（二）多措并举提品质增优势，农业增效农民增收成效明显

种植业结构不断优化。充分发挥有为政府和有效市场作用，推动种植结构合理调整，粮食作物综合效益明显提升。大豆种植面积10年来首次超过玉米，由98.3万亩调增到228.3万亩，上调了132.2%。玉米面积由365.9万亩调减到225.8万亩，下调了38%。水稻面积由119万亩扩大到132.6万亩，上调了11.4%。杂粮杂豆、食用菌等高值高效作物面积扩大到30.2万亩。农产品营销成效显著。农民市场意识和营销能力显著增强，各类农业经营主体外销农产品达到124.5万吨。营销方式日趋多元化，线上营销、展会营销已成为重要渠道。省大米网双鸭山“一馆四城”建成，在深圳“农迈天下”、“深商E天下”等知名电商平台建立了双鸭山农产品网站，线上交易量增长35%。参加各类展会15次，签约量1.1万吨。加大与深圳、佛山农产品供销合作力度，深圳、佛山分别建立了双鸭山农产品交易中心和直营店，与深农、深粮等大企业建立了长期合作关系，仅深农集团旗下的海吉星商城、上海农产品批发中心、南昌农产品批发中心就订购了我市11.2万吨富硒大米等农产品。珠三角地区已经成为我市农产品重要外销地，“双宝谷坊”、“田地冰米”等品牌深受当地消费者青睐。参加省“农担杯”优质农产品营销大赛获第4名。绿色生态农业加快发展。建设高标准种植示范基地46个，“三减”示范面积达到61万亩，绿色食品基地认证面积达到136.6万亩。绿色食品产品认证数量达到69个，地理标志产品达到10个。实施农田水利工程项目13个，有效提高了农业综合生产能力。宝清县、饶河县被评为全国水稻绿色高产高效创建示范县，集贤县绿色食品示范基地被列为省级农业科技园区示范基地。农村改革继续深化。深入实施“两大平原”综合配套改革，农村集体产权制度改革试点、土地“三权分置”稳步实施，完成417个行政村土地确权实测工作。大力培育新型经营主体，推动了农民合作社由生产主体向经营主体加快转变，促进了一二三产融合发展。全市规范合作社达到220家，生产经营型合作社达到40家；完成家庭农场认定310个；土地规模经营面积达到260万亩。

一年来，我们以促进农业增效农民增收为目标，狠抓种植结构调整、生态农业建设和市场营销，农业综合生产能力和市场竞争力明显增强，有力推动了现代农业融合发展。

（三）持之以恒抓改革促开放，经济发展活力有效释放

重点领域改革取得扎实成效。行政审批制度改革方面，加大“放管服”力度，全面推行了“一站式”网上审批，行政审批事项全省最少；非行政许可审批事项全部取消；实施了“多证合一、一照一码”商事登记制度。经济体制改革方面，推进了国有资产管理体制改革、盐业体制改革、国有林场改革等重点领域改革，厂办大集体改革基本完成。综合执法体制改革方面，推行了“双随机、一公开”监管方式；实现了食药安全网格化监管体系全覆盖；城管执法和文化市场综合执法均实现了行政处罚权集中行使。社会管理体制改革方面，启动了区域规划体系改革、智慧城市建设及管理体制改革；实施了不动产登记制度、公立医院人事薪酬制度、教育职称评聘制度改革；将全市93个公共服务热线整合到110指挥平台；完成了统筹城乡的居民基本医疗保险整合；建立了多元化民生服务体系和就业服务体系；社区盖章证明事项由90项精简到8项。推行“大科室制”，调整了14个经济综合管理部门职能和内设机构。对外交流合作向纵深发展。“双深合作”形成了机制化、常态化合作的良好态势，合作领域和深度不断拓展，项目建设、粮食销售、电商、文化、体育、卫生等领域合作不断深化，已落实合作项目13个，正在推进合作项目97个。“两山合作”全面启动，两市政府正式签订了对口合作框架协议，明确了产业建设、项目对接、园区共建、绿色食品销售等8个合作领域，一批产业项目和各领域合作正在有序推进。全市组织各类招商活动106次，举办了第二届双鸭山经贸合作企业家恳谈会，形成招商线索181项。参加中央电视台“魅力中国城”竞演活动，荣获“魅力城市”称号，提升了双鸭山的知名度和影响力。以对俄合作为重点的对外开放不断扩大，预计实现外贸进出口总值1.5亿美元，同比增长6%。

一年来，我们把改革开放作为加快发展的重要举措，以深化重点领域改革释放发展活力，以全面提升开放水平拓展发展空间，为转型发展注入了新的强大动力。

（四）尽心竭力办实事惠民生，人民群众获得感显著增强

大力改善居民生活条件，让群众生活更加美好。全面完成27768套采沉区棚改任务，煤矿棚改工程竣工住宅7970套，宝山区采沉区综合治理避险安置一期开工住宅3453套，改造农村泥草房1799户。投入1亿多元，改造了主城区73个老旧住宅小区、581栋住宅楼，12万户居民居住环境得到极大改善。主城区实现了物业管理全覆盖。全面改造主城区二次加压供水泵站，供水二次加压费全部取消。完成了窑地路、马鞍山路等11.9公里供水管网和260栋老旧楼房供水管道的新建和改造。免费为14811户老旧小区居民更换了室内供水管道，为3万户居民更换了智能水表。完成了主城区34.1公里老旧供热管网改造。新建、改扩建主城区公共卫生间18个。全面启动了6个独立工矿区和4个区本部“两供一业”综合改造。引进天然气战略投资者，南市区天然气置换顺利启动，标志着我市即将进入“清洁燃气时代”。启动了中心站立交桥建设，建设路立交桥、南环路开工建设，北外环路、石材路、花菇路建成通车，打通了龙翔小区、富丽小区“断头路”，整治背街巷道“疮疤”路 651条。我市被中央文明委纳入全国文明城市（提名城市）创建序列。着力提升公共服务水平，让群众生活更有品质。全市投入10亿元，大力改善办学条件，新建、改扩建三十二中、二中等义务教育阶段学校、公办幼儿园32所，6个县区顺利通过国家义务教育均衡发展验收。整合职教资源，职教集团办学水平有新提升。市人民医院投入使用，市中医院改造完成，市精神病防治院、老年人精神病福利院、6个养老机构主体工程完工。公共文化服务体系建设迈出新的步伐，文化产业发展取得新的进展，市图书馆新馆开工建设，市图书馆南市区分馆完成主体工程，6个“三室”活动中心建成。加快立体交通网建设，让群众出行更加便捷。牡佳客专双鸭山段施工顺利，高铁片区总体规划加快编制。尖山区至宝山区公路、饶河县至大岱林场公路建设顺利，集贤县至友谊县公路开工建设。省政府重点给予宝山区至宝清县一级公路资金支持，前期工作正在有序推进。积极推动重新启动了双佳机场项目前期工作，宝清、饶河机场项目前期取得实质性进展。以高速铁路、高速公路、高等级公路、机场为支撑的立体交通格局逐步形成。持续提高社会保障水平，让群众享受更多发展成果。全市城镇新增就业2.27万人。加大对双矿转岗职工就业创业扶持力度。医疗保险普惠面和待遇大幅提高，大病医疗保险调整为大额医疗保险，城镇职工、城镇居民、新农合居民年度内最高支付限额分别达到20万元、11万元和28万元，在全国范围内实现了异地就医直接结算。城乡居民基本养老金标准比上年提高14%。城市低保标准提高到每月540元，农村低保标准提高到每年3780元。发放救助资金4.7亿元，困难职工基本生活得到有效保障。全力推进脱贫攻坚，让困难群众过上好生活。精准识别建档立卡贫困户1737户、3363人。大力推进精准扶贫，实施扶贫产业项目120个。加大金融扶贫力度，投入政府风险补偿金2075万元、发放扶贫小额贷款7506万元。强化政策保障兜底，“两不愁、三保障”问题不断有效解决。

一年来，我们积极践行以人民为中心的发展思想，从群众最期盼、最急需的问题抓起，着力补短板、兜底线、惠民生，切实把民生实事办到人民群众的心坎上。人代会上承诺的50项民生实事全部完成年度计划，已完成39项，11项正在按计划有序推进。

（五）坚持不懈转职能优服务，政府管理和服务水平持续提升

社会管理不断强化。突出抓好以煤矿为重点的安全生产，全面推行安全生产网格化和“一项制度、三个清单”的安全监管机制，深入开展“打非治违”和“隐患排查整治”专项行动，各类生产安全事故同比下降40%。全面推行“四个一批”治访模式，有效解决群众合理合法诉求，全市信访形势持续好转，荣获“全国信访先进单位”称号。扎实推进“平安双鸭山”建设，完成“天眼工程”三期视频监控系统建设，严厉打击各类违法犯罪活动，实现治安、刑事案件发案双下降。依法行政深入推进。自觉接受人大法律监督和政协民主监督，办结人大代表建议50件、政协委员提案115件，办结率100%。深入开展“七五”普法。全面推行了法律顾问制度。社会诚信体系建设取得成效。扎实推进“两学一做”学习教育常态化制度化。认真落实党风廉政建设责任制，健全权力运行制约监督体系，强化行政监察和审计监督，深入开展作风整顿，营造了风清气正的发展环境。生态保护和监管扎实有效。持续推进造林绿化、山体修复、节能减排和污染防治，建龙干熄焦项目建成试运行，城市生活垃圾无害化处理项目已确定投资主体，再生水利用工程完成调试，淘汰建成区小锅炉90台、黄标车1527台。全面实行“河长制”，一些重要的基础性工作走在全省前列。开展了安邦河流域水环境综合整治专项行动和保护母亲河行动，已完成治理项目16个。全市环境空气质量达标率为90.6%。市级城市集中式饮用水水源水质达标率为100%。

市场监管、外事侨务、民族宗教、防灾减灾、国防动员、统计、文史档案等各项工作及妇女儿童、老龄和残疾人事业都取得了新的进展。

各位代表，一年来，我们牢固树立“四个意识”，团结全市上下，汇聚发展合力，坚决有力地贯彻落实党中央国务院、省委省政府和市委各项决策部署；我们牢牢把握发展第一要务，积极践行新发展理念，着眼于打基础、增后劲、利长远，谋划推动了一批关系全局和发展的大事；我们本着对双鸭山发展和人民群众高度负责的态度，敢于直面矛盾，勇于担当尽责，创新突破，攻坚克难，有效解决了一批事关发展、稳定、民生的难题和历史遗留问题；我们始终坚持以人民为中心的发展思想，真情回应群众期盼，办成了一批涉及群众切身利益的民生实事；我们大力弘扬真抓实干的工作作风，着力强素质、转作风、提效能，夙兴夜寐，激情工作，用实干、实绩、实效推动了各项事业发展。

成绩来之不易，创业充满艰辛。我们深切体会到，双鸭山发展取得的每一个成就、发生的每一处变化、人民群众得到的每一点实惠，都得益于省委、省政府和市委的坚强领导，得益于市人大和市政协的有效监督和鼎力支持，更得益于全市上下的拼搏实干，凝聚着全市人民的辛勤汗水。在此，我代表市人民政府，向全市人民，向各级人大代表、政协委员，向各民主党派、工商联、无党派人士和人民团体，向驻双解放军、武警官兵和公安干警，向在双中省直单位和所有关心支持双鸭山发展的各界人士，表示衷心的感谢!

在总结成绩的同时，我们也清醒认识到，发展中还存在很多困难和问题。主要是：新经济总量偏小，民营经济活力不足，产业转型升级任重道远；民生保障能力和水平还不够高，满足人民群众对美好生活需要的任务依然艰巨；市场化程度不高，体制机制不活，改革力度仍需加大；政府职能转变还不到位，一些部门工作水平和办事效率低下，部分干部创新意识不强，工作标准和专业化水平不高，高效服务的意识和能力有待提高。对此，我们将以对人民、对事业高度负责的态度，采取更加有效、更加务实的措施予以解决。

二、2018年主要任务

2018年，是贯彻党的十九大精神的开局之年，是在我市步入经济社会发展新阶段的背景下，加快转型发展、决胜全面建成小康社会、实施“十三五”规划承上启下的关键一年。市政府工作的总体要求是：以习近平新时代中国特色社会主义思想为指导，全面贯彻党的十九大精神、中央经济工作会议精神、省委十二届二次全会精神和市委十一届二次全会精神，坚持新发展理念，坚持稳中求进总基调，坚持以供给侧结构性改革为主线，以改革创新为动力，以提高保障和改善民生水平为根本，统筹推进稳增长、促改革、调结构、惠民生、防风险各项工作，抓重点、补短板、强弱项，打好转方式调结构、精准脱贫、重要领域改革、污染防治、防范化解重大风险、整顿作风优化发展环境的攻坚战，加快推进“四区一城”建设，大力发展“七大主导产业”，加快经济社会转型发展步伐，为决胜全面建成小康社会夯实基础，为建设富强民主文明和谐美丽的现代化双鸭山而不懈努力。2018年发展的主要预期目标是：全市地区生产总值增长6%；规模以上工业增加值增长4%；固定资产投资增长9%；公共财政预算收入增长6%；外贸进出口总值增长5%；社会消费品零售总额增长7%；城镇和农村常住居民人均可支配收入均增长7%；节能减排完成省定指标。

（一）着力建设现代产业体系。加快转型发展，必须坚持一产抓融合、二产抓提升、三产抓拓展，以提高供给体系质量为主攻方向，加快推动接续替代产业集群化发展，打造接续替代产业升级版。必须锁定新增长领域这个主攻方向。加快调整产业结构，向资源开发和精深加工要发展，向优势产业和产品延伸升级要发展。全市上下必须把培育新增长领域作为主攻方向和战略抓手，摆上经济工作的核心位置，采取超常规举措，强力组织推进，确保现代煤化工、粮食和绿色食品深加工、石墨及新材料、旅游、电商、畜牧、特色种植七个新增长领域取得实实在在的成果。必须以产业项目作支撑。在现代煤化工方面，推动龙煤天泰10万吨芳烃、三聚华本5万吨LNG等项目竣工投产，加快落实煤制烯烃、煤制乙二醇等项目投资主体。在粮食和绿色食品深加工方面，加快集贤县威克特10万吨特优食用酒精、宝清县万里润达150万吨玉米深加工、集贤县鸿展30万吨特优食用酒精等项目建设，启动3个省定点区域玉米燃料乙醇项目建设。在石墨及新材料方面，加快岭东区中双石墨采选及深加工项目建设，加快羊鼻山、西山、西沟等区块石墨资源勘查，启动石墨产业发展规划编制。在畜牧产业方面，在推动集贤县大北农百万头生猪一体化项目开工的同时，再引进几个较大规模的生猪养殖、饲料加工及畜产品深加工项目。在旅游产业方面，以打造“中国·黑土湿地之都湿地旅游带”和“乌苏里江·界江风情旅游带”为牵动，着力在发展黑土湿地旅游和乌苏里江风情旅游新业态上实现新进展，在打造饶河县四排乡赫哲族民俗特色风情旅游小镇和房车自驾游营地上取得新突破。在电商产业方面，按照“模式升级、发展加速、产业壮大”的思路，在完善平台功能、打造电商品牌、电商企业孵化等方面发力，推动电商产业提档升级。在特色种植产业方面，通过科技引领、龙头带动、规模发展，加快形成食用菌产业集群、林业产业集群、棚室蔬菜种植产业集群，努力把小产品发展成大产业。全年建设500万元以上产业项目155个，其中5000万元以上项目52个，完成投资75亿元。必须加大园区建设力度。市经开区要加快我市与佛山市共建经济合作园区进度，并以此为抓手，加大基础设施建设力度和项目引进力度，确保园区建设质量和项目引进及管理水平有明显提升。根据战略投资者引进工作情况，适时启动现代煤化工产业园区建设。四个县的产业园区都要在完善园区功能、强化基础设施、引进投资项目上下功夫。必须激发市场主体活力。要立足我市本地现有市场主体，通过理念的更新、观念的转变、环境的优化、政策的引导和激励措施的应用，进一步激发本地企业家创新创业激情和活力，弘扬企业家精神，使优秀企业家成为全社会创新创业的典型和榜样。设立专项资金，启动实施“企业家培训计划”，全年计划培训1000名企业家。要坚持引资、引技、引模式、引渠道相结合，加大盘活存量企业和新企业生成力度，尽快形成一批新的市场主体。必须强化区域协调发展。牢固树立全市“一盘棋”思想，整合区域发展优势，要在政策、服务等方面给予农垦、森工、双矿更大支持，充分调动各功能主体的积极性和创造性，激发发展活力；推动农垦、森工、双矿与市、县（区）融合发展，加快形成特色鲜明、优势明显的主导产业，形成优势互补、协调并进、共同繁荣的区域发展格局。按照政策要求和上级部署，积极做好企业办社会移交的承接工作。要注重推动四个区的整体协调发展，逐步解决在发展上的不平衡不充分的问题。

（二）着力实施创新驱动发展战略。加快转型发展，必须把工作的基点放在创新上。科技创新是城市竞争力的核心，是推动产业发展、经济转型的根本力量。全市上下必须把科技创新牢牢地抓在手上，向高新技术成果产业化要发展。深化与省内外高等院校、科研院所的合作，鼓励和推动高等院校、科研院所在我市设立研发机构、建立科技成果转化基地。完成省科学院在我市设立科技成果转化中心、东北农大设立科研机构工作。围绕加快推动科技创新、产业发展、经济转型，加大引进“外脑”工作力度。继续选派优秀干部到高等院校和科研院所培训锻炼，“提高科学认识、学习科技知识、结识科技专家、引进科技成果”。2018年，要实现四县四区、市经开区与高等院校、科研院所建立产业科技创新联盟全覆盖。实施重点科技成果转化和科技研发项目20项，力争培育科技型中小企业30家、一定规模科技型企业8家。大众创业万众创新是发展动力之源、是富民之道。要加快市、县区电商产业园、电商产业基地模式升级、功能完善，把电商产业园、电商产业基地打造成“双创”的重要载体。加快创业孵化器、科技孵化器和众创空间建设，强化“双创”平台支撑。整合市创业指导中心、市人才服务中心等4个中心及职能，成立市“双创”指导中心，强化“双创”指导功能，提升服务水平。设立专项资金，实施“双创培训计划”，重点对高校毕业生、城镇转岗职工、城镇再就业人员、农民工和退伍军人等群体进行培训，提高他们的创业就业技能和本领。加大政策扶持力度，采取免费为创业者提供棚改闲置商服等措施，推动创业者进入新领域、发展新业态、运用新模式创新创业。全年实现城镇居民成功创业2000人、农民成功创业2000人。智慧城市既是推动城市治理能力现代化的重要抓手，又是承载推动新经济、新业态发展的共享平台。要根据“双鸭山市智慧城市建设方案”总体设计，采取PPP模式启动建设全市统一的智慧城市运营中心、数据资源中心和智慧政务、智慧公共安全、智慧城管、智慧教育、智慧医疗、智慧诚信、智慧旅游、智慧农业等14个应用系统，有效支撑政府管理和服务效能的提升。同时，还要推动“互联网+”在更广阔领域应用，打破信息壁垒，促进技术融合、业务融合、数据融合，构建促进新模式、新业态、新产业创新发展的资源共享体系和大数据平台。

（三）着力实施乡村振兴战略。加快转型发展，必须坚持农业农村优先发展。要以深化农业供给侧结构性改革为主线，按照产业兴旺、生态宜居、乡风文明、治理有效、生活富裕的总要求，加快推进农业农村现代化。把产业兴旺作为乡村振兴的基础。要以构建现代农业产业体系、生产体系、经营体系为抓手，加快发展现代农业。大力优化农业种植结构。扩大高值高效粮食种植面积，增加特色经济作物面积，特别是要下大力气抓好棚室蔬菜、食用菌种植和林业经济。“互联网+农业”高标准种植示范基地达到50个，农产品质量安全实现可追溯。深入推进“三减”，扩大绿色食品供给，绿色食品基地认证面积发展到140万亩。加强农田基础设施建设。农机作业综合机械化程度达到95.5%；新增高效节水灌溉面积1万亩、“旱改水”面积1万亩，加快大叶沟水库和“引松入双”工程前期工作。加快推进一二三产融合发展。以现代农业园区、农产品加工企业和新型经营主体为牵动，促进农业生产、加工、物流、服务等相互融合，促进农业全环节升级、全链条增值。各县要在现代农业园区建设上有新进展，各乡镇要在三次产业融合发展上有新实践。要延伸价值链，把“卖得好”摆在突出位置，培育特色品牌，强化市场营销。把深化农村改革作为乡村振兴的关键。要把培育新型经营主体作为重要工作，加快推动农民合作社、家庭农场由单一生产型向生产经营型转变。生产经营型合作社达到60个以上。加快农村土地“三权分置”改革，全面完成农村土地确权，土地流转面积达到230万亩以上。大力推进农村集体产权制度改革，完成全市农村集体资产清产核资工作和60个村改革任务。把生态宜居作为乡村振兴的重要任务。以开展农村人居环境整治三年行动为抓手，以农村垃圾、污水治理和村容村貌提升为主攻方向，加快建设以“六个美”为目标的美丽乡村。建立分层投入机制，启动农村“厕所革命”整村推进试点，解决农村如厕难、洗澡难、排污难问题。注重建立健全自治、法治、德治相结合的乡村治理体系，培养造就一支懂农业、爱农村、爱农民的“三农”工作队伍。

（四）着力深化重要领域改革。加快转型发展，必须坚决打好重要领域改革攻坚战，破除制约发展的体制机制弊端。深化经济体制改革，激发市场活力。深入推进以“放管服”为核心的简政放权。深化行政审批制度改革，进一步优化审批流程。规范完善权力清单、责任清单、中介服务清单。全面实施市场准入负面清单制度，清理废除妨碍统一市场和公平竞争的规定和做法。深化商事制度改革，推进“多证合一、一照一码”及“证照分离”改革。支持民营企业发展，加快竞争性经营行业和投资领域向民营企业开放。推进不动产登记机构集成模式改革，做到“一窗受理、集成服务”。实行国税地税联合办税改革。深化机构和行政体制改革，进一步转变政府职能。统筹考虑政府系统各类机构设置，科学配置部门及内设机构权力，明确职责，推进政府职能由管理型向服务型转变。统筹使用各类编制资源，形成科学合理的管理体制。推进经营类事业单位改革，实行政事分开、事企分开、管办分离。深化社会管理体制改革，提高治理能力。深化医疗卫生体制改革，加快五项基本医疗卫生制度建设，加大公立医院综合改革力度。继续推进教育系统人事制度改革，深化教育职称评聘制度改革，构建社会、家庭、学校“三位一体”大教育格局。深化食品药品监管体制机制改革，完成城市综合执法行政处罚权集中行使改革。推动“双随机、一公开”监管方式全覆盖。推动市容市貌管理立法。加快建立多主体供应、多渠道保障、租购并举的住房制度。建立完善守信联合激励和失信联合惩戒制度，加强政务诚信体系、个人诚信体系、电商领域诚信建设。深入推进公共法律体系建设，推进公证体制改革。探索建立城市有效管理的新机制，推进供热、供水、污水、垃圾处理、物业管理等领域市场化配置资源改革。推动金融体制改革，释放金融发展活力。推动建立市级金融工作议事协调机制，成立地方金融监管局，加强属地金融监管。推动地方金融业改革，完成4家农信社改制为农商行的改革任务。引进省鑫正投资担保公司，在我市建立担保平台。打好防范化解重大风险攻坚战，增强金融风险管控能力，规范和控制政府举债，防止发生系统性区域性金融风险。

（五）着力构建对外合作交流新格局。加快转型发展，必须走开放之路、合作之路，向开放要活力，向引进外来战略投资者要发展。扩大以对俄为重点的对外开放。按照省委“一窗四区”部署，全力打造黑龙江东部对俄合作产业聚集区，加强和完善口岸功能，以发展跨境物流、跨境电商为纽带，加快农产品、木材、矿产资源进出口加工基地和物流基地建设，推动对俄合作由经贸领域向全方位合作拓展。推动中俄经贸产业园物流区、饶河县锦隆物流保鲜库建成运营，做实做优饶河县中俄互市贸易区、宝清县白瓜籽大市场、岭东区花菇基地、宝山区“爱喜爱”宠物食品加工等一批进出口基地。加强与国内发达地区的合作交流。抓住我省与广东对口合作的重大机遇，巩固提升“双深合作”水平。“两山合作”力求在产业项目开发、粮食和绿色食品营销、旅游、金融、科技交流、共建园区等领域取得阶段性合作成果，努力把“两山合作”打造成为跨区域合作的典范和标杆。同时，要拓展与“珠三角”、“长三角”和“环渤海”等发达地区的有效合作与交流。加大招商引资力度。把高质量策划生成项目作为招商引资的重要基础和前提，全年策划生成5000万以上市场对接成功率高的项目60个；充分依托我市资源优势、产业基础，实现与有意图扩大投资规模、实现产业梯度转移的大型央企、国企、民企精准对接；创新招商引资模式，通过引进新经济、新业态、新模式，打造新的招商引资平台。推动宁波蓝源资本集团“众农联”特色产业链平台型总部基地项目落地。

（六）着力加强生态文明建设。加快转型发展，必须坚持人与自然和谐共生，坚决打好污染防治攻坚战，加快美丽双鸭山建设。坚持绿色发展理念。加强山水林田湖草及湿地生态系统保护和修复，推进治水、净气、降噪、还绿、护田及垃圾处置“六大工程”，完成生态保护红线划定，编制重点生态功能保护区规划，严守生态保护红线、永久基本农田、城镇开发边界三条控制线，逐步将我市的生态优势转化为经济发展优势。坚决打赢蓝天保卫战。加大重点企业排放达标治理力度和监管力度，完成国电、大唐热电超低排放改造，完成虹焱热电、饶河县晨光热电、宝清县热电联产项目达标治理，强力推进20蒸吨及以上供热锅炉和电力锅炉达标排放治理，对重点污染排放源实施在线自动监测。加大淘汰小锅炉、黄标车力度。坚持疏堵结合，以“五化”为主攻方向，加大秸秆综合利用推进力度，重点在能源化上下功夫，秸秆综合利用率达到40%。加大水污染防治力度。严格落实“河长制”，加强污染防治，保护水环境。加大城市母亲河——安邦河流域水环境综合整治力度，继续推进6大类28项重点任务实施，加快安邦河水系生态化治理进度。加大主城区和集贤县雨污分流改造和再生水利用推进力度。加强农业面源污染综合治理。开工建设城市生活垃圾无害化处理项目。加强生态环境监管。落实落靠环境保护主体责任，严格执行生态环境损害责任终身追究制。制定自然资源资产管理和生态环境保护考核办法，严格执行领导干部自然资源资产离任审计制度。加大“城市双修”推进和督查力度。申请调整饶河县东北黑蜂、宝清县七星河、集贤县安邦河等国家级、省级自然保护区范围，加强对各类保护区的保护和监管。将农垦、森工、双矿纳入全市生态环境监管联防联治体系，开展环境监察多部门联合执法，加大环境违法行为查处力度，严厉打击环境违法犯罪。

（七）着力提升城市承载力。加快转型发展，必须以创建全国文明城市为契机，加快补齐城市短板，完善城市功能。城市规划在城市发展中起着重要引领作用。要高度重视城市总体设计修编工作，加快调整完善城市总体规划，进一步增强规划的科学性、前瞻性、系统性和引领性。要坚持“全市一盘棋、规划一张图”，统筹经济社会发展规划、城乡规划、土地利用规划等专项规划，构建多规合一、系统完备的规划体系。完成城市设计，启动重点工矿区总体规划编制。开工建设集规划、科技、智慧城市展示于一体的城市规划科技馆。建设立体交通网是打破交通瓶颈制约的重要举措。全力推进牡佳客专双鸭山段征地拆迁，确保工程建设顺利进行。按照零换乘的思路，完成牡佳客专双鸭山西站和市客运中心一体化设计。推动双佳机场前期工作，力争宝清和饶河机场前期工作取得突破性进展。开工建设大岱林场至红旗岭和集贤镇至福利镇公路，加快友谊县至集贤县公路建设，确保尖山区至宝山区公路、饶河县至大岱林场公路竣工通车。全力推进全长82公里的贯通我市东西的宝山区至宝清县一级公路开工建设。完善城市功能必须加快城市基础设施建设。要在继续推进城市建设“六大工程”实施的基础上，确保在六个方面取得新进展：加大道桥建设力度，确保建设路立交桥竣工通车，加快中心站立交桥建设,开工建设公立村立交桥；完成城市南环路、康泰路、岭东区滨水路建设和窑地路、岭东区东矿路与青山路改扩建工程；继续推进主城区背街巷道改造和交通微循环工程。加大老旧小区改造力度，修缮改造16个小区132栋住宅楼，全面完成主城区老旧小区改造任务；全面完成6个独立工矿区和4个区本部“两供一业”综合改造。加快天然气置换工程建设，确保2018年完成5万户居民的天然气置换。加大老旧管网改造力度，新建、改造供热管网44.9公里、供水管网12公里、燃气管网41.5公里、雨污分流主管网18公里，全面完成供热、供水一级二级管网改造。加大绿化美化亮化力度，提升新兴大街、双鸭山火车站等城市主要节点和沿街楼体亮化水平，完成世纪大道及民生路绿化改造提档升级工程，完成城市东部山体市民公园二期工程。加大公共服务设施建设力度，推动第三十二中学、第四中学、第二中学等5所学校加快建设并投入使用，新建、改扩建公办幼儿园5所；完成口腔医院搬迁改造、妇幼保健院改造并投入使用；开工建设南市区体育训练运动综合馆；完成市图书馆新馆建设，市图书馆南市区分馆投入使用。加大城市建成区违法建设专项治理力度，坚决遏制新增违法建设，存量违法建设查处比例累计达到50%以上，加快改善人居环境，营造良好的生态、生活、生产空间。

（八）着力抓好脱贫攻坚。加快转型发展，必须坚决打好精准脱贫攻坚战，全面完成集贤县、友谊县、宝清县脱贫任务，做好饶河县退出国家级贫困县迎检工作。确保全程精准。要聚焦“六个精准”，在确保扶贫对象精准、资金使用精准、因村派人精准的基础上，突出项目安排精准，做到每个贫困户至少有一个产业项目带动增收；突出措施到位精准，因户施策、一户一策；突出脱贫成效精准，严把退出关，做到脱真贫、真脱贫。落实好政策。要聚焦“两不愁、三保障”，确保落实到位。在教育保障方面，要把各学段贫困学生补助资助政策落实落靠，让每一名贫困学生上得起学、上得好学。在医疗保障方面，要全面落实贫困人口医疗保障救助政策。特别是要高度关注因病、因残致贫占比高这个最难啃的“硬骨头”，拿出更加务实管用的办法，确保这部分贫困人口吃得起药、看得起病，托住政策这个底，防止脱贫之后再度返贫。在住房保障方面，要全面完成贫困户危房改造，让贫困户住上安全房、暖屋子。同时，要完成贫困人口安全饮水改造，完成贫困村“三通三有”建设。突出抓好产业扶贫。重点扶持贫困户发展特色种植、养殖和庭院经济等效益好的产业项目。扶持贫困村发展符合本村特点、符合一二三产融合发展的产业和光伏产业，带动贫困户增收脱贫。强化贫困户与农产品加工企业、农民合作社或村集体的联结机制，在产业发展中设计合理的利益分配机制，通过发展集体经济实现村民共同富裕。用好用足扶贫小额贷款，真正发挥扶贫贷款的扶贫效益。压实落靠责任。压实县区主体责任和主要领导负总责的责任制，落靠各级扶贫开发办及相关部门、结对帮扶单位和驻村工作队的工作责任，锁定目标，聚力攻坚，坚决打赢脱贫攻坚这场硬仗。

（九）着力提高保障和改善民生水平。加快转型发展，必须牢固树立以人民为中心的发展思想。就业是最大的民生。要坚持就业优先战略，实施更加积极的就业政策，完善公共就业服务平台，大规模开展职业技能培训，坚持以创业带动就业，多渠道开发就业岗位，推进重点群体就业创业，全年开展职业技能培训8000人，实现城镇新增就业2万人、转移农村劳动力13万人，促进城乡居民收入水平不断提高。教育是面向未来的事业，是每个家庭都关注的问题。巩固已通过国检的7个县区义务教育均衡发展成果，推动宝山区通过国家验收。要制定全市教育教学质量提升计划，通过优化师资力量配备、开展教师教学培训，提升教师教学水平，抓好素质教育，全面推动全市教育教学质量的进一步提高，办好人民满意教育。办好学前教育，加强幼儿园规范管理，普惠性幼儿园覆盖率达到40%。抓好职业教育，以市场需求为导向，以提高教学和培训质量为核心，加快把职教集团打造成高质量的技能人才培训基地。加强师德师风建设，大力整治教育“三乱”。改善居住条件是人民群众的期盼。全面完成煤矿棚改工程和采沉区棚改工程的配套工程。加大宝山区采沉区综合治理工程推进力度，完成一期3453套住宅建设。启动15211户城市棚户区改造工程。年底前已建成的所有棚改工程全部完成回迁安置。社会保障是民生安全网、社会稳定器。全面实施全民参保计划，促进人人享有基本社会保障。做好养老保险全国统筹前期准备工作。完成机关事业单位养老保险制度改革。全面实施统一的城乡居民基本医疗保险和大病保险。扎实做好社会救助、临时救助，保障城乡困难群众基本生活。积极发展残疾人事业。推进健康双鸭山建设事关每个人的工作生活和家庭幸福。完成全市医联体调整和重新布局，加强基层医疗卫生服务体系建设和全科医生队伍建设。加大重大疾病防控力度。支持社会力量办医，发展健康养老产业。深入开展爱国卫生运动，通过省级卫生城市复检验收。广泛开展“三减三健”行动。严格落实食品药品安全监管责任，抓好“双安双创”推进工作，确保食品药品安全。文化体育事业发展是衡量美好生活的重要标志。深入实施文化惠民工程，加强基层文化设施建设，推动文艺精品创作；广泛深入开展群众文化活动，推进高雅艺术进入校园。加大文物保护力度，加强非物质文化遗产保护传承。强化文化市场管理，推动文化产业发展。广泛开展全民健身运动，深入推进“三大球”和冰雪运动项目进校园，发展冰球、速滑、滑雪等冰雪体育运动和冰雪产业。推动学校和社会体育场馆资源共享。

改善和提高生活水平始终是群众所盼、我们的职责所系。必须大力推动产业发展、推动大众创业万众创新，千方百计提高人民收入水平，使改革发展成果惠及全市人民。城镇和农村常住居民人均可支配收入均增长7%的目标要力保完成。同时，要落实好相关政策，从2018年1月份起，按工资总额8%的标准兑现市本级机关事业单位在职和离退休人员的住房提租补贴，让大家有更多的获得感。

（十）着力加强和创新社会治理。加快转型发展，必须把推进社会治理体系和治理能力现代化作为重要内容。安全生产事关人民群众福祉，事关经济社会发展大局。要强化属地管理，严格落实“党政同责、一岗双责、齐抓共管、失职追责”；强化行业、部门监管责任，严格落实“三个必须”；强化企业主体责任，做到安全责任、管理、投入、培训和应急救援“五到位”。要建立企业风险管控和隐患排查治理双重预防机制，严格落实安全生产网格化和“一项制度、三个清单”常态化监管机制。要狠抓煤矿安全生产，持续推进“打非治违”和“专项整治”，严格排查整改隐患，坚决遏制重特大事故发生。要认真落实省委巡视组巡视整改意见，在2018年年底前完成地方煤矿兼并重组和整治整合任务，完成省下达的淘汰落后产能工作任务。狠抓非煤矿山、危险化学品、道路交通、建筑施工等各行业领域安全隐患排查整改，确保安全生产形势持续稳定。社会治安防控体系是社会治安综合治理的主要依托。要深入实施公安系统“六大建设工程”，在全市推开“龙防云”系统，加大以天眼工程为重点的技防网络建设力度，提高社会治安数字化防控水平，大力整治治安突出问题，严厉打击各类违法犯罪活动，切实保障广大人民群众人身权、财产权、人格权，提高人民群众的安全感和满意度。加强信访工作是有效预防和化解社会矛盾的内在要求。要严格落实信访工作责任制，逐级夯实属地责任、部门责任和领导责任，构建有责要担当、失责必追究的信访工作责任体系。坚持问题导向，事要解决，把合理合法的诉求解决到位，努力把矛盾化解在萌芽状态，把问题解决在基层。坚持依法治访，按照“三个到位、一个处理”的要求，坚持“四个一批”治访模式，解决信访工作中遇到的突出问题，推动信访工作向制度化、法治化方向迈进。社区是社会治理的基础平台。要以建设“幸福社区”为载体，加快推进社区活动由面向少数人向大多数人转变，社区服务由被动服务向主动服务转变，社区工作由机关化服务向深入千家万户服务转变。2018年，要按照“六统一”标准，完成10个集文化、体育、教育、卫生、养老日间照料、残疾人康复六项服务功能于一体的“幸福社区”综合活动室试点建设。

三、加强政府自身建设

加快转型发展，为全面建成小康社会奠定坚实基础，为建设现代化双鸭山积蓄力量，是新时代和全市人民赋予各级政府的艰巨任务。必须坚决打好整顿作风优化发展环境攻坚战，增强凝聚力、激发创造力、强化执行力、提高战斗力，把各级政府建设成人民满意的服务型政府。

（一）要大力解放思想。解放思想是解决一切问题的“总开关”。加快转型发展，实现全面振兴，必然要面临许多新情况、新问题，特别是我市正处于爬坡过坎、滚石上山的关键阶段，面临着许多困难和问题，一些长期积累的矛盾已经到了必须解决的程度。各级政府和广大干部必须大力解放思想，坚决革除思想僵化、因循守旧，畏难怕险、无所作为，激情不足、自甘落后等思想障碍，要跳出固有思维，树立全新思维，以新的理念、新的视野谋划发展、推动工作，善于从改革中找动力、从市场中找出路、从创新中激发活力，加快汇聚起推动转型发展的强大动能。

（二）要增强专业本领。干部的能力和水平要从学习中来。各级政府和广大干部必须把加强专业知识学习摆在突出位置，在学习上下一番真功夫、苦功夫、实功夫，特别是要加强对经济、科技、管理等领域新理念新知识的学习和国家政策的学习，加快知识更新、优化知识结构，着力克服本领不足、本领恐慌、本领落后的问题。要继续办好“双鸭山发展讲坛”，执行好政府系统专业知识专题学习制度，使学习专业知识成为一种常态、一种自觉、一种风气，切实增强专业本领，提高开展工作、解决问题、推动转型的专业化水平。

（三）要强化责任担当。担当是一种责任，更是一种能力。加快转型发展，实现全面振兴，重任在肩，不容懈怠。各级政府和广大干部必须在状态敢担当、善攻坚有作为，坚决革除责任意识不强、担当精神不足的问题，特别是遇到矛盾绕着走、碰到难题往后退的问题，以敢打必胜的信心和决心，以不达目的不罢休的干劲和韧劲，以等不起、慢不得、坐不住的紧迫感、危机感和责任感，攻坚克难，奋勇拼搏，推动转型发展、全面振兴取得经得起历史、实践和人民检验的成果。

（四）要狠抓工作落实。一分部署，九分落实。各级政府和广大干部必须真抓实干，高效率、高标准推进转型发展、全面振兴各项工作任务落实，坚决革除工作效率低下、粗枝大叶、标准不高，只求过得去、不求过得硬等顽症固疾，对部署的工作、定下的事情，坚决执行、迅速落实、马上就办、一抓到底，使夙兴夜寐、激情工作成为一种常态；要切实践行“工匠精神”，做到掌握情况、分析问题、制定方案、配套措施、工作落实都要细，在精细化中出彩，一级做给一级看、一级带着一级干，以工作落实的实际成效检验我们的工作成果、体现我们干部的工作能力和水平。

（五）要加强作风建设。作风建设永远在路上。各级政府和广大干部要始终把作风建设牢牢地抓在手上，坚决执行《中央八项规定实施细则》，弛而不息整治“四风”；坚决整治慢作为、粗作为、不作为、乱作为的问题；坚决查处败坏作风、破坏发展环境的人和事，让歪风邪气无处遁形，让我们这座城市始终充满公平正义的正能量。各级政府和领导干部要始终把廉政建设主体责任扛在肩上、抓在手上，做到廉政建设与政府工作同步研究、同步推进、同步落实。要强化制度约束，把权力关进制度的笼子里，加大对重大事项决策、大额资金使用、政府投资项目、公共资源交易、资本运作等重点领域和关键环节廉政风险防控力度，深入推进正风反腐。

各位代表，使命呼唤担当，实干成就梦想，实现2018年工作目标任务，需要全市上下勠力同心，加倍努力。让我们紧密团结在以习近平同志为核心的党中央周围，在省委省政府和市委的正确领导下，不忘初心、牢记使命，与全市人民一道攻坚克难、务实创新、奋发进取，携手创造双鸭山加快转型发展新业绩，共同谱写人民美好生活新篇章！
